# The Moralizing Effect: self-directed emotions and their impact on culpability attributions

**DOI:** 10.3389/fnint.2023.1232523

**Published:** 2023-11-28

**Authors:** Elisabetta Sirgiovanni, Joanna Smolenski, Ben Abelson, Taylor Webb

**Affiliations:** ^1^Section of History of Medicine, Department of Molecular Medicine, Sapienza University of Rome, Rome, Italy; ^2^Center for Medical Ethics and Health Policy, Baylor College of Medicine, Houston, TX, United States; ^3^School of Liberal Arts, Mercy College, Dobbs Ferry, NY, United States; ^4^Department of Psychology, University of California, Los Angeles, Los Angeles, CA, United States

**Keywords:** guilt, shame, self-directed emotion, responsibility, culpability, morality, judgment

## Abstract

**Introduction:**

A general trend in the psychological literature suggests that guilt contributes to morality more than shame does. Unlike shame-prone individuals, guilt-prone individuals internalize the causality of negative events, attribute responsibility in the first person, and engage in responsible behavior. However, it is not known how guilt- and shame-proneness interact with the attribution of responsibility to others.

**Methods:**

In two Web-based experiments, participants reported their attributions of moral culpability (i.e., responsibility, causality, punishment and decision-making) about morally ambiguous acts of killing in different conditions. In Study 1 the vignettes were presented in the 1st person, while in Study 2 in the 3rd person. To test proneness to guilt and shame, we utilized the GASP scale, which differentiates between the affective and behavioral components of each emotion. Statistical analyses were performed in Matlab and R.

**Results:**

We found that guilt- and shame-proneness were associated with the severity of attributions in both the first and the third person, but the effect was strong only in the guilt case (both subtypes) and shame-affect case, and not in the shame-behavior case. We call this the Moralizing Effect.

**Discussion:**

We wonder whether our finding that guilt-prone people tend to attribute a higher degree of culpability to others is really consistent with the view that guilt motivates people to choose the “moral paths in life”. This echoes views about the harmful aspects of guilt, which have been expressed historically in philosophy, for example, by Friedrich Nietzsche.

## Introduction

Psychological research on moral judgment has recently renewed interest in complex, self-directed emotions (e.g., guilt and shame). These emotions had been neglected for a couple of decades (Prinz, 2011). In fact, after some seminal research in the 1990s (see Tangney and Dearing, 2002 for a review), moral psychology had focused mainly on basic, other-directed emotions (e.g., disgust, anger, and contempt, see Rozin et al., 1999; Haidt, 2003; Wheatley and Haidt, 2005; Schnall et al., 2008; Pizzarro et al., 2011; Seidel and Prinz, 2013). Guilt and shame are both negative, painful emotions one experiences when transgressing against some moral rule (Tangney et al., 2007). They have been shown to co-occur and laypeople tend to not consistently distinguish between the two terms (Tangney and Dearing, 2002, Ch. 2). However, psychology (Keltner and Buswell, 1996; Tangney et al., 1996) and neuroscience (Michl et al., 2014; Bastin et al., 2016) have provided evidence that they are distinct emotions. Accordingly, some theoretical distinctions between guilt and shame have been adopted in empirical research. These include the idea that guilt is directed toward behavior while shame is toward the self (Lewis, 1971), and that guilt occurs when one’s moral infractions remain private, while shame is caused by public display of one’s transgressions (Benedict, 1946). The two emotions have also been associated with characteristic behavioral responses: reparative behavior for guilt and avoidant behavior for shame, respectively (Cohen et al., 2011). Excessive guilt and shame are also included as symptoms of many psychiatric conditions (e.g., depression, hypomanic episodes, post-traumatic stress disorder, dissociative disorders, eating disorders, sexual dysfunction, borderline personality disorder, and paraphilic disorder for guilt; obsessive-compulsive disorder, post-traumatic stress disorder, dissociative disorders, eating disorders, borderline, avoidant disorder, and anxiety for shame) (American Psychiatric Association, 2013), while individuals with antisocial traits have been shown to lack these two emotions (Hare, 1998), especially guilt. Nevertheless, guilt and shame are not always distinguished consistently in the DSM (see Fontenelle et al., 2015).

Indeed, there is a general trend in the psychological literature suggesting that guilt contributes to morality more than shame does. Remarkably, there are divergent meanings of what “*being* moral” might denote, for instance either acting in a certain way (i.e., being a moral *agent*) or distinguishing right from wrong (i.e., being a moral *judge*) - and it is not so obvious that one sense should entail the other as traditional moral philosophers believed (Cova, 2013). In this area of research about guilt and shame, morality has been intended mostly in agentive rather than reflective terms, i.e., in this context morality has meant pro-sociality. In June Tangney and collaborators’ words, “empirical results converge, indicating that guilt but not shame is most effective in motivating people to choose the moral paths in life” (Tangney et al., 2007, p. 10). The narrative of guilt being more moral than shame has developed largely due to research on the relationship between guilt/shame and behavioral intentions concerning hypothetical scenarios (i.e., participants responded to questions like “*how would you behave in such situation?*”), and less often explicitly on the relationship between guilt/shame and moral judgments of the rightness/wrongness of actions (for an overview, Tangney and Dearing, 2002; see also Cohen et al., 2012). In support of the claim about the agentive morality of guilt vs. shame, Tangney (1994) found evidence of a positive correlation between self-reported moral behavior with guilt, but not with shame. Guilt in general seems to enhance one’s sense of self-control and risky judgments (Kouchaki et al., 2014) and positively correlates with forgiveness-seeking, but only in the absence of shame (Riek et al., 2014). Moreover, high guilt-prone individuals engage in counterproductive work behavior less frequently (Cohen et al., 2012, 2013), have better leadership skills (Schaumberg and Flynn, 2012), and are more likely to be interpersonally trustworthy at the workplace (Levine et al., 2018), than low guilt-prone individuals. By contrast, shame, but not guilt, has been linked to anger and self-reported aggression (Tangney et al., 1992a,b), as well as criminal behavior and recidivism (Tangney et al., 2011, 2014). It must be noted, however, that in most of these experiments, the actions correlating with guilt and shame were hypothetical and self-reported. Some correlations, (e.g., between shame and criminal behavior) were based on arrest records.

Speculative attempts at characterizing potentially harmful aspects of guilt were engaged in by the philosopher Friedrich Nietzsche in his second essay of *On the Genealogy of Morality* (1887), “‘Guilt’, ‘bad conscience’ and related matters.” Nietzsche thought that guilt, being an inward cruelty, turned into a *bad conscience*, which is potentially a result of the internalization of outward moralization (particularly, that associated with the Christian tradition) created by *ressentiment* towards others, an attitude that he views negatively. In his own words, “it is easy to guess who has the invention of ‘bad conscience’ on his conscience, − the man of *ressentiment*!” (Nietzsche, 1887/2006, p. 49, original italics; for a discussion see Elgat, 2017). These ideas, elaborated by Freudian psychoanalysis (e.g., Freud, 1923; see also Piers and Singer, 1953; on Nietzsche and Freud see Greer, 2002; see also Jervis, 1998), have been discarded by empirical psychology as they were thought to conflate guilt and shame, thereby mislabeling shame experiences as guilt experiences (Lewis, 1971; Tangney and Dearing, 2002). Some traces of these ideas, however, remain in clinical psychopathology, where guilt is believed to play not only a “social-adaptive role,” but to be psychologically “destructive” at an intrapsychic level, or in other words, excessively self-punitive (Carnì et al., 2013 for an overview). Deontological guilt, a form of guilt elicited by having disobeyed an intuitive moral rule, was shown to lead to ethically questionable decisions such as failure to flip the switch in the standard trolley dilemma (Mancini and Gangemi, 2015), or acceptance of unfair offers in ultimatum game tasks (Mancini and Mancini, 2015). Other recent experiments, such as those showing that guilt makes people excessively focus their attention on the victim of their behavior at the expense of others, have similarly questioned the morality of guilt (de Hooge et al., 2011). A few authors have speculated in defense of shame, for example, as a “guardian” of self-identity and integrity (Deonna et al., 2012). However, the general narrative that shame, but not guilt, can be deleterious is deep-rooted in psychology and philosophy (see Nussbaum, 2009).

Moreover, one might suppose that divergent moral outcomes result from the link, presumed by Nietzsche scholars and Freudians, between guilt/shame and different religious traditions, or from the link between these emotions and different cultures, as proposed by some anthropologists (Benedict, 1946). These ideas have inspired only a few, inconclusive, empirical studies (e.g., Hutchinson et al., 1998; Walinga et al., 2005; Vaisey and Smith, 2008; see also Tangney and Dearing, 2002, pp. 152–153).

However, guilt and shame are not only significant for their contribution to pro- or anti-social behaviors. Experts in the field tend to agree that when these emotions are present, “some degree of moral judgment would seem to be indirectly involved” (Tangney & Dearing, 2002, p. 43) and that “one has internalized moral values” (Cohen et al., 2012, p. 355). In the debate about guilt and shame, these emotions are considered “‘self-conscious emotions’ that are evoked by self-reflection and self-evaluation” (Tangney and Tracy, 2013), whether “implicit or explicit, consciously experienced or transpiring beyond our awareness,” directed alternatively toward the behavior or toward the self (see also Cohen et al., 2011). Although moral judgment is not necessarily indicative of moral action (Cova, 2013), our research was driven by the idea that it is worthwhile to understand the moral judgments that guilt/shame prone individuals make, so as to better characterize the connection between these emotions, beliefs and the kind of actions they motivate. Indeed, this assessment of the “antecedents” of moral behavior may be a first step in addressing a question about morality that has been taken to be relevant to past empirical research on moral emotions in general (Ellemers et al., 2019).

In order to be morally accountable, an agent need not possess a sophisticated knowledge of her moral reasons (i.e., so-called *deliberative agency*, see Levi and Bayne, 2004), but at least she has to recognize the conditions under which one, including herself, might be held responsible or punished for some transgressions. This is a minimal solution to problems such as automatism or involuntary conduct, which has been suggested in the legal domain (Yannoulidis, 2012), and is easily applicable to morality.

We broadly define prosocial or antisocial emotions, respectively, as those that elicit behavior that may benefit or hurt others, including society as a whole (see Gilbert and Basran, 2019). It is worth noting that in order to differentiate between the prosocial or antisocial nature of an emotion, we focus on the behavior, rather than on the emotion itself. To characterize guilt and shame as pro-social or anti-social, we consider the benefit or harm to others that would result from their effect on moral judgment. It must also be noted that the idea of equating morality with mere pro-sociality is contested by influential moral philosophers. For example, according to John Harris, morality is conceived as “basically a matter of choosing what is for the best all things considered, not simply being well motivated or pro-social; in short that to be good is not simply happening to do no evil but choosing for a reason, choosing on the basis of evidence and argument, not to do wrong” (Harris, 2016a, p. 270; see also Harris, 2016b). This position contests the view defended by some neuropsychologists that emotional interventions aimed at increasing pro-sociality, bypassing cognition, would be sufficient to enhance someone’s moral behavior.

Additionally, guilt and shame are intimately related to the notion of responsibility - indeed, ‘guilty’ and ‘responsible’ are often used as synonyms in ordinary language. Given that responsibility attributions are a significant metric for investigating people’s moral beliefs (Nichols and Knobe, 2007), investigating this relationship can give us insight into the moral beliefs of guilt- and shame-prone individuals and contribute to the analysis of the morality of these emotions. Thus far, attributions of responsibility have only been tested in the context of guilt and shame in the first-person dimension (Tangney, 1990; Tangney et al., 1996; Riek et al., 2014). The links with attributional styles (in terms of causality and blame) have also been addressed, but only when the scenarios were presented in the first person. It was found that guilt tends to result in the internalization of cause or blame for negative events (self-blame), while shame results in their externalization (Tangney, 1990; Tangney et al., 1992a,b). The relation between guilt/shame and responsibility attributions in the third-person dimension, the existence of which seems less obvious than in the first-person, has not been explicitly investigated. Specifically, although it is known that guilt-prone individuals make severe attributions of responsibility to themselves, we do not know whether they will also make such severe judgments about others. A large body of previous work has highlighted asymmetries between first and third person judgements, such as the actor-observer asymmetry (Jones and Nisbett, 1971), the self-serving bias (Miller and Ross, 1975), or the fundamental attribution error (Ross, 1977), all evidencing more benevolent judgments in the first person than in the third. However, it is not known whether and how these asymmetries will interact with guilt- and shame-proneness.

Another open question concerns the distinct factors that contribute to responsibility attributions in folk ethics. People usually believe, to hold someone responsible for an action, the someone in question must have caused the outcomes of an action (causality), decided intentionally to act that way (decision-making), and be consistently punishable for that action (punishment) (Fischer and Ravizza, 1998). These other dimensions, relevant to responsibility, are different, separate components that contribute to responsibility attributions in degrees and have not been investigated together by previous studies. Among the three, punishment attributions concern a self-reported action to be taken in the future (i.e., to punish or not), so they are related to agentive morality exactly as most previous literature on guilt/shame investigates. Thus, considering punishment alongside with other antecedent conditions (causality, decision-making) may suggest an interesting relationship between guilt/shame and moral action/judgment.

The case for investigating causality and decision-making separately is based on the well-established fact that people sometimes tend to judge negatively harm caused even unintentionally (Cushman, 2008). We must note that, even in folk ethics, there are apparently exceptions to the presence of causality (i.e., omissions) and decision-making (i.e., negligence) for responsibility attributions. However, it seems that even in these exceptional cases causality and decision-making are somehow present and relevant. In omissions, one just performs a *different action* from the action one should perform as prescribed by social norms, while in negligence one still decides and intends to act in a certain way even if one somehow ignores the *consequences* of that action.

To this end, the objective of our study was to see whether and how guilt and shame impact responsibility attributions in all their specific dimensions that precede and follow the action, including causality, decision-making, and punishment. Moral *culpability* (Moore, 1996) seems to be the concept that properly brings together responsibility, causality, decision-making, and punishment, in the guilt dimension, given that *culpa* is the Latin word for guilt.

We tested the severity of culpability attributions both in the first- and third-person for individuals who are prone to guilt or shame. Apart from examining the first/third person dimension, we also explored the impact of: (a) different types of interconnected attributions, all related to the broader concept of moral culpability (i.e., responsibility, causality, decision-making, and punishment) (see for example Hitchcock and Knobe, 2009 for the link between norms and causality); (b) the introduction of mitigating conditions (such as coercion) and (c) affective and behavioral components of guilt and shame, using a recently introduced trait assessment, the GASP scale (Cohen et al., 2011) that differentiates between these subcomponents.

We hypothesized that culpability attributions would be more severe in guilt-prone individuals than in shame-prone individuals. In the psychological literature, amplifying the moral significance of violations when making moral judgments has been named *moralizing* (Rozin, 1999; Horberg et al., 2009, 2011). Consistent with this literature, we refer to severe culpability attributions as moralizing. We also hypothesized that, consistently with the attributional style patterns found for guilt and shame by previous studies (Tangney, 1990; Tangney et al., 1992a,b), showing that guilt internalizes causality and blame of actions while shame externalizes them, the effect would be more pronounced in the first-person dimension than in the third for guilt-prone individuals (*self-critical* moralizing, or a form of internalization of the blaming). Alternatively, we expected more severity in the third-person than in the first for shame-prone individuals (*other-critical* moralizing, or a form of externalization of the blaming).

## Materials and methods

We performed two Internet-based experiments to test whether proneness to guilt and/or shame affects culpability attributions. The research, including its materials and methods, was approved by the Institutional Review Board at the City University of New York. Formal permission for using the Guilt and Shame Proneness Scale (Cohen et al., 2011) was provided by the American Psychological Association and by the first author of the scale.

The two surveys, composed of 5 sections totaling 27 questions, were designed through the SurveyMonkey platform. English-speaking participants were recruited through Amazon Mechanical Turk. While we are aware that online crowdsourcing platforms may have several limitations, among them, MTurk has been shown to obtain quality and representative data, especially within the United States, and is also an especially diverse subject pool (Mason and Suri, 2012; Strickland and Stoops, 2019).

All participants expressed written informed consent at the beginning of each survey (Section 1, Q1). After being assigned a code, they were compensated with a small sum (0.50 USD) for their participation at the end of the survey.

We collected a sample of online participants in both studies 1 and 2. The number of participants who completed the survey in Study 1 were *N =* 101, including 48 women, 52 men and 1 subject who did not identify with either category. Participants’ ages ranged from 19 to 69 years old (*M =* 35.48). A hundred participants were from the United States, and only one was from elsewhere.

In Study 2, *N* = 116 participants completed the survey, including 56 women, 57 men, and 3 individuals who did not identify with either of these categories. Participants’ ages ranged from 18 to 75 years old (*M =* 35.4), and all participants were from the U.S. Information regarding the ethnicity and religious affiliation of all participants in both studies can be found in Table 1.

**Table 1:** Demographic information for all participants in Study 1 (*N* = 101) and Study 2 (*N* = 116).

**A.**		
	Study 1	Study 2
African American, Black	10	11
Chinese	3	2
Indian	2	2
Japanese	0	1
Korean	3	1
Southeast Asian	1	1
White Caucasian - Non Hispanic	50	63
Hispanic or Latino	7	3
Mexican	1	1
American Indian, Alaskan Native	0	0
Middle Eastern	1	0
European, White	19	24
More than one race	1	3
Not reported or listed	1	2
Decline to answer	2	2

(B)				
	Study 1 (RA - childhood)	Study 1 (RA - current)	Study 2 (RA - childhood)	Study 2 (RA - current)
Protestant	24	17	40	25
Orthodox	1	2	4	3
Catholic	37	22	27	18
Islamic	2	3	1	0
Jewish	2	2	2	3
Buddhist	0	1	1	2
Hindu	0	0	1	1
Confucian	0	0	0	0
Yes (religious), not listed	10	13	11	14
None (Atheist, Agnostic, etc.)	23	39	26	44
Decline to answer	2	2	3	6

**(A)** Ethnic and racial background. **(B)** Religious affiliation (RA - childhood and current).

To test attributions of moral culpability, we designed 4 different vignettes, each of which described the same morally ambiguous act of killing under different conditions. These vignettes were presented randomly to participants in Section 2 (Q2-5) just after expressing informed consent. In each of the 4 scenarios, the agent finds a stranger at home and fatally stabs and kills this intruder. Three of the vignettes include a different potentially mitigating circumstance (i.e., intoxication, coercion, and false information), and the 4th, a control vignette, had no such circumstance (see Table 2). Due to the conceptual vagueness of the term ‘responsibility,’ we decided not to ask only for explicit responsibility attributions, but also for other kinds of attributions often conceptually connected to the notion of responsibility, all belonging to the broader concept of moral culpability (see Fischer and Ravizza, 1998). The 4 questions (i.e., about responsibility, causality, decision-making, and punishment) are listed in Table 3. The participants indicated their responses on a 7 point Likert-type scale (from 1 = *not at all*, to 7 = *strongly*).

**Table 2:** Moral vignettes in different conditions (blank, intoxication, coercion, false information) and kinds of questions (responsibility, causality, punishment, decision-making) for 1st/3rd person version.

**Blank condition** 1st/3rd person version *You (Sam) come(s) home at night to find(s) a stranger standing in your living room. You (Sam) see(s) a sharp kitchen knife on the counter, pick(s) it up and fatally stab(s) the stranger in the chest.*
**Intoxication condition** 1st/ 3rd person version *You (Sam) come(s) home at night intoxicated from an evening of heavy drinking and find(s) a stranger standing in your living room. You (Sam) see(s) a sharp kitchen knife on the counter, drunkenly pick(s) it up and fatally stab(s) the stranger in the chest*.
**Coercion condition** 1st/3rd person version *You (Sam) come(s) home at night to find two strangers standing in your living room. One is unarmed but the other has a gun. The one with the gun orders you (Sam) to pick up the sharp kitchen knife on the counter and stab the unarmed stranger and says that if you (Sam) fail(s) to comply you (Sam) will be shot in the head. You (Sam) pick(s) up the knife and fatally stab(s) the unarmed stranger in the chest.*
**False information condition** 1st/3rd person version *On your way home one night you (Sam) receive(s) an anonymous text message telling you (Sam) that there is a stranger in your (Sam’s) apartment who means to kill you (Sam). When you (Sam) get(s) home there is, indeed, a stranger standing in your (Sam’s) living room. You (Sam) see(s) a sharp kitchen knife on the counter, pick(s) it up and fatally stab(s) the stranger in the chest. However, in fact the text you (Sam) received was a lie. The stranger in your apartment meant you (Sam) no harm and was actually there seeking safety from the person who sent the text.*

**Table 3:** Moral vignettes in different conditions (blank, intoxication, coercion, false information) and kinds of questions (responsibility, causality, punishment, decision-making) for 1st/3rd person version.

**Questions for 1st/3rd person version**
*- How responsible are you (is Sam) for performing this action?* (responsibility question) *- How much did you (Sam) cause this outcome?* (causality question) *- How much should you (Sam) be punished?* (punishment question) *- Did you (Sam) decide to perform this action?* (decision-making question)

In Study 1, the agent in the vignettes was presented in the 2nd person (“you”), asking the participant to put him/herself in the scenario in order to obtain 1^st^ person attributions. In Study 2, the agent, for whom we chose the gender-neutral name “Sam,” was presented in the 3^rd^ person.

To test for individual proneness to guilt and shame, we used all 16 items of the Guilt and Shame Proneness Scale (GASP) (Cohen et al., 2011). The GASP scale was built and validated in continuity with previous scales (e.g., TOSCA-3, Tangney et al., 2000). However, we decided to use the GASP scale because, unlike the TOSCA (see Giner-Sorolla et al., 2011), the GASP differentiates between the affective and behavioral components of each emotion, resulting in 4 subscales. Specifically, the GASP scale contains 4 items for guilt-negative behavior evaluation (Guilt-NBE), 4 items for guilt-repair (Guilt-R), 4 items for shame-negative self-evaluation (Shame-NSE) and 4 items for shame-withdraw (Shame-W). Guilt-NBE items are meant to capture a person’s proneness to bad feelings about her actions, while Guilt-R items are concerned with one’s proneness to intend to compensate for her actions. Meanwhile, Shame-NSE items address a person’s proneness to feeling bad about herself after a transgression, while Shame-W has to do with proneness to intend to hide or withdraw. Our choice to keep the affective and behavioral components of guilt and shame distinct was motivated by an interest in whether or not the components differentially affect responsibility attributions. To this end, we decided to look at trait emotions rather than induced emotions since the induction of guilt and shame separately, especially the induction of their specific subtypes, would be very difficult and would imply a choice among different kinds of inductions (McLatchie et al., 2016).

In the GASP, participants are asked to answer questions about their likelihood, on a 7 point Likert-type scale from 1 (=*unlikely*) to 7 (=*very likely*), to feel or act in a particular way, which is intended to measure their possession of characteristics corresponding to the 4 subscales (examples of the GASP items are in Table 4).

**Table 4:** Four sample items of the GASP scale (Cohen et al., 2011), one for each subscale.

Sub-scale	Sample items
**Guilt-NBE**	*After realizing you have received too much change at a store, you decide to keep it because the salesclerk does not notice. What is the likelihood that you would feel uncomfortable about keeping the money?*
**Guilt-repair**	*You reveal a friend’s secret, though your friend never finds out. What is the likelihood that your failure to keep the secret would lead you to exert extra effort to keep secrets in the future?*
**Shame-NSE**	*You rip an article out of a journal in the library and take it with you. Your teacher discovers what you did and tells the librarian and your entire class. What is the likelihood that this would make you would feel like a bad person?*
**Shame-withdraw**	*After making a big mistake on an important project at work in which people were depending on you, your boss criticizes you in front of your coworkers. What is the likelihood that you would feign sickness and leave work?*

We tested participants using the GASP scale (Section 3, Q6–Q21) after asking them the four types of attribution questions in response to the moral vignettes (Q2-5). We used a 7 point Likert-type scale for attributions to be consistent with the range of responses to the GASP items. We chose to present the moral vignettes before the GASP scale in order to avoid any possible influence of the GASP items. In principle, it is possible that the moral vignettes affected responses to the GASP scale. It would be useful in future work to determine whether and how the order of presentation affects the observed relationship between these two measures.

Demographic questions (Section 4, Q22–Q27) were posed at the end, asking for gender, ethnic/racial background, country of residence, current religion, childhood religion, and age. Internal randomization was utilized in all 3 sections.

Statistical analyses were performed in Matlab and R.

## Results

We first examined the extent to which moral culpability, characterized by the 4 types of attributions (responsibility, causality, punishment, and decision-making), is predicted by scores on the GASP’s 4 subscales for guilt and shame (descriptive statistics for these variables are presented in Table 5).

**Table 5:** A. Descriptive statistics for all independent variables (GASP subscales). B. Descriptive statistics for all dependent variables.

	Mean	St. Dev.	Range	α
Guilt-NBE	5.30	1.35	1–7	0.81
Guilt-Repair	5.49	1.10	1–7	0.73
Shame-NSE	5.37	1.27	1–7	0.78
Shame-Withdraw	3.49	1.28	1–7	0.65
α indicates the internal reliability of each subscale.				

	Mean	St. Dev.	Range
Responsible	4.93	1.36	1–7
Causal	4.71	1.38	1–7
Punish	3.81	1.48	1–7
Decision	5.16	1.37	1–7

To do this, we produced a single attribution score for each participant by taking the average of all attributions made by that participant. We then performed 4 separate linear regressions, each examining the predictive capacity of 1 subscale. We followed the GASP authors’ recommendation to perform separate regression analyses in order to avoid possible multicollinearity problems and to retain any shared variance between guilt and shame that might be excluded by creating the artificial constructs of guilt-free shame and shame-free guilt (Cohen et al., 2011, p. 955). We did so as we concurred with the GASP authors in their skepticism about the “utility of such computations given that, phenomenologically, people are unlikely to experience guilt without a hint of shame or shame without a tinge of guilt” (Cohen et al., 2011, p. 951). This decision is justified statistically by the fact that guilt and shame are shown to typically co-occur (Paulhus et al., 2004).

**Figure 1:**
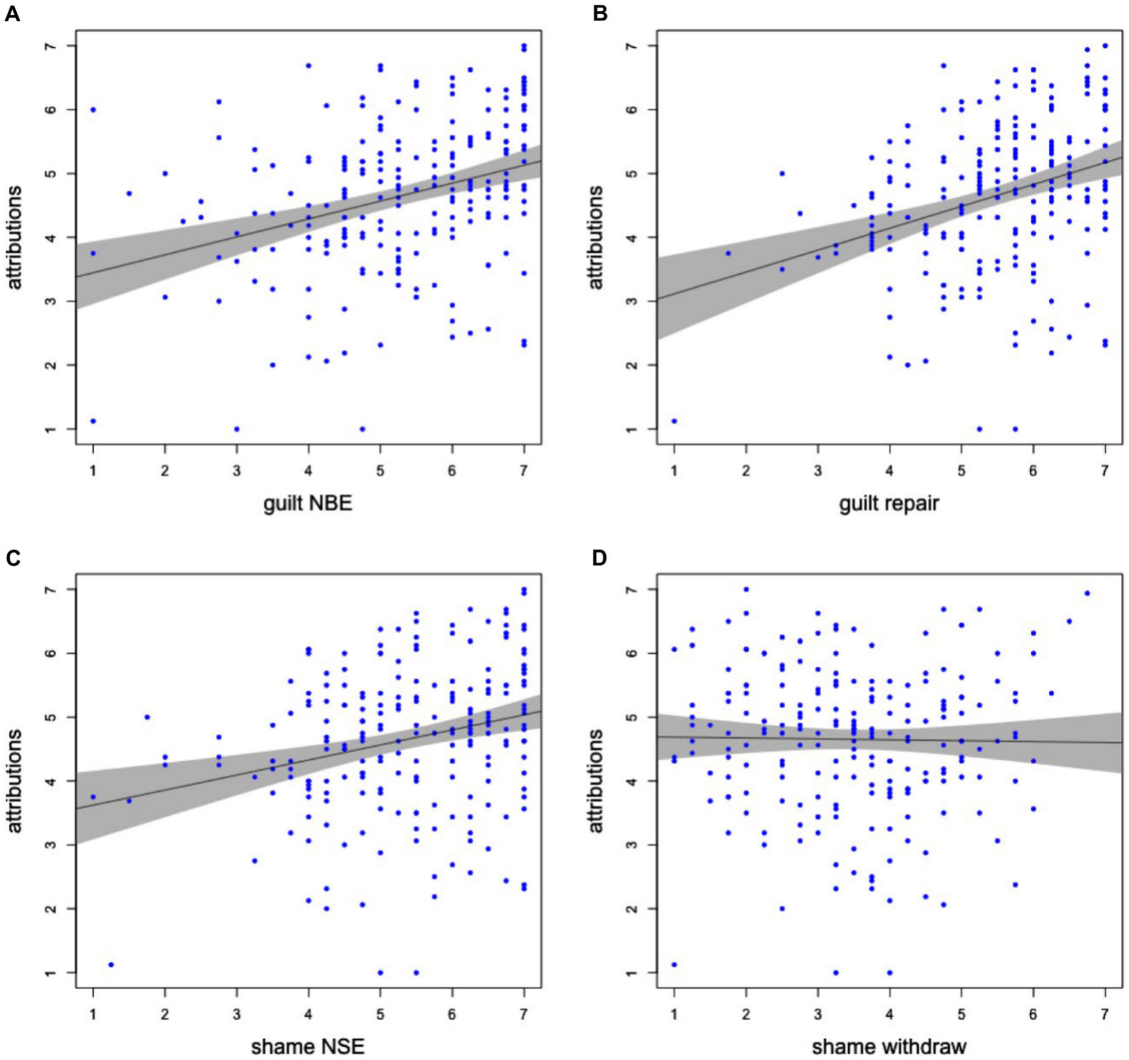
Regression models for the relationship between culpability attributions and subscales for guilt and shame. Shaded gray area represents 95% confidence intervals. **(A)** Affective subscale for guilt (Guilt-NBE). **(B)** Behavioral subscale for guilt (Guilt-R). **(C)** Affective subscale for shame (Shame-NSE). **(D)** Behavioral subscale for shame (Shame-W).

Because 4 separate regressions were performed, we performed Bonferroni corrections for 4 comparisons. The results revealed that attributions were significantly predicted by the subscales for both the affective dimension of guilt (Figure 1A, *β* = 0.28, *p* < 0.0001, Bonferroni corrected for 4 comparisons) and the behavioral dimension of guilt (Figure 1B, *β* = 0.34, *p* < 0.0001, Bonferroni corrected for 4 comparisons), but only the affective dimension of shame (Figure 1C, *β* = 0.24, *p* < 0.001, Bonferroni corrected for 4 comparisons). The subscale for the behavioral dimension of shame did not significantly predict attributions of any type (Figure 1D, *β* = −0.01, *p* = 0.81, uncorrected). Interestingly, this result mirrors the covariance structure reported by the scale authors, whereby the affective subscale for shame (Shame-NSE) shared more variance with the guilt subscales (i.e., Guilt-NBE and Guilt-R) than it did with the behavioral subscale for shame (Shame-W) (*Ibid.*, p. 950). These results are consistent with our moralizing effect hypothesis for both dimensions of guilt and the affective dimension of shame, but not the behavioral one.

We next examined whether this pattern was different when participants were making 1st vs. 3rd person attributions. We performed 8 separate regressions, assessing the extent to which the 4 subscales predicted attributions separately in the 1st and 3rd person conditions. We did not find any evidence to support our hypotheses regarding differences between 1st and 3rd person attributions, and, indeed, the pattern looked remarkably similar in these two conditions. In the 1st-person condition, attributions were significantly predicted by both the affective (Guilt-NBE, *β* = 0.26, *p* < 0.05, Bonferroni corrected for 8 comparisons) and behavioral (Guilt-R, *β* = 0.41, *p* < 0.01, Bonferroni corrected for 8 comparisons) dimensions of guilt, and by the affective dimension of shame (Shame-NSE, *β* = 0.33, *p* < 0.05, Bonferroni corrected for 8 comparisons), but not the behavioral dimension of shame (Shame-W, *β* = −0.1, *p* = 0.36, uncorrected). In the 3rd-person condition, attributions were significantly predicted by both the affective (Guilt-NBE, *β* = 0.3, *p* < 0.001, Bonferroni corrected for 8 comparisons) and behavioral (Guilt-R, *β* = 0.3, *p* < 0.01, Bonferroni corrected for 8 comparisons) dimensions of guilt. The results were marginally significant for the affective dimension of shame, but did not pass Bonferroni corrections (Shame-NSE, *β* = 0.18, *p* = 0.0125, uncorrected), and were not significant for the behavioral dimension of shame (Shame-W, *β* = 0.05, *p* = 0.46, uncorrected). We also looked for interactions between the 4 subscales and the 1st vs. 3rd person dimension. Each regression included the one dependent variable, our average measure of culpability attributions, and three independent variables: (1) 1 of the 4 subscales for guilt and shame, (2) whether the moral attributions in question were 1st or 3rd person (coded as a dichotomous, between-subjects predictor), and (3) the interaction between variables 1 and 2. These results were Bonferroni corrected for 4 comparisons. The results revealed no significant interactions between 1st vs. 3rd person attributions and the subscales for guilt and shame, and, surprisingly, no main effect of 1st vs. 3rd person (Table 6A). To confirm this, we performed a t-test between 1st and 3rd person attributions, which again revealed no significant effect (two-sample *t*-test, *t* = 0.82, *p* = 0.41). In sum, we did not find any evidence in support of our hypotheses regarding ways in which guilt and shame proneness might interact with 1st vs. 3rd person attributions, and instead found a pattern that was robust across these two conditions.

**Table 6:** Regression models for interactions between the subscales for guilt and shame and different contexts of culpability attribution.

**(A)**						
	Subscale		First/third person		Interaction	
	*β*	*p*	*β*	*p*	*β*	*p*
Guilt – affective	0.26	< 0.01	−0.04	N.S.	0.03	N.S.
Guilt – behavioral	0.41	< 0.001	0.76	N.S.	−0.11	N.S.
Shame – affective	0.33	< 0.01	0.99	N.S.	−0.15	N.S.
Shame - behavioral	−0.11	N.S.	−0.43	N.S.	0.16	N.S.

**(B)**						
	Subscale		Mitigating factors		Interaction	
	*β*	*p*	*β*	*p*	*β*	*p*
Guilt – affective	0.25	<0.01	−0.87	N.S.	0.04	N.S.
Guilt – behavioral	0.38	<0.0001	−0.38	N.S.	−0.05	N.S.
Shame – affective	0.26	<0.01	−0.47	N.S.	−0.03	N.S.
Shame – behavioral	−0.0004	N.S.	−0.59	N.S.	−0.02	N.S.

*P-*values are Bonferroni corrected for four comparisons, unless noted otherwise. N.S. (not significant) indicates predictors with *p* > 0.05 after correction for multiple comparisons. **(A)** Interaction with first vs. third person attributions. **(B)** Interaction with mitigating factors.

Finally, we examined the effect of factors that might mitigate culpability attributions. These included circumstances in which agents acted under conditions of coercion, intoxication, or false information, as well as a control case that did not involve mitigating factors. We found the same pattern when considering only cases that included mitigating factors. Limiting the data to these cases, we found that attributions were significantly predicted by both the affective (Guilt-NBE, *β* = 0.3, *p* < 0.0001, Bonferroni corrected for 4 comparisons) and behavioral (Guilt-R, *β* = 0.33, *p* < 0.0001, Bonferroni corrected for 4 comparisons) dimensions of guilt, and the affective dimension of shame (Shame-NSE, *β* = 0.23, *p* < 0.01, Bonferroni corrected for 4 comparisons), but not the behavioral dimension of shame (Shame-W, *β* = −0.02, *p* = 0.76, uncorrected). We also looked for interactions between the 4 subscales and the presence of mitigating factors. We performed 4 separate multiple regressions. The dependent variable for each regression was our average measure of culpability attributions, and the independent variables were: (1) 1 of the 4 subscales for guilt and shame, (2) whether the scenario in question involved mitigating factors (dichotomous, within-subjects predictor), and (3) the interaction between variables 1 and 2. These results revealed no significant interactions between the subscales for guilt and shame and whether an attribution involved mitigating factors (Table 6B). Overall, these analyses showed that the primary pattern of results, that attributions are predicted by both subscales of guilt, and by the affective but not behavioral dimension of shame, was robust to the presence of potentially mitigating factors.

Surprisingly, our analysis did not reveal a significant main effect of mitigating factors, suggesting that perhaps participants were not sensitive to these factors in making culpability attributions. To investigate further, we performed two analyses. First, we directly compared culpability attributions in cases with vs. without mitigating conditions. This analysis yielded a significant result (paired *t*-test, *t* = 9.56, *p* < 0.0001). This result suggests that the lack of a main effect of mitigating factors is due in part to the presence of the interaction term in the regressions in Table 6B. Without this term, the effect of mitigating factors is significant. Second, we examined each type of mitigating factor separately. We performed paired t-tests between attributions involving 1 of the 3 different kinds of potentially mitigating factors, and attributions involving no mitigating factors. Because we asked participants about scenarios involving 3 different kinds of mitigating factors, we performed Bonferroni corrections for 3 comparisons. This analysis revealed that attributions of culpability were significantly altered by scenarios involving either coercion (paired *t*-test, *t* = 13.5, *p* < 0.0001, Bonferroni corrected for 3 comparisons) or false information (paired *t*-test, *t* = 3.5, *p* < 0.01, Bonferroni corrected for 3 comparisons), but not intoxication (paired *t*-test, *t* = 0.9, *p* = 0.34, uncorrected). This result suggests that the lack of a main effect of mitigating factors (in the regressions reported in Table 6B) is due in part to the fact that participants found some mitigating factors (intoxication) less exculpating than others (coercion and false information). In sum, though we do not find any evidence for an interaction between guilt/shame and the presence of mitigating factors, our data do suggest that the presence of certain mitigating factors independently affects culpability attributions.

As for possible demographic factors (i.e., gender, childhood religion, current religion) we found only that women tend to be more Guilt-NBE prone than men (two-sample *t*-test, *t* = 3.1, *p* < 0.01, Bonferroni corrected for 4 comparisons). Given this disparity, we also tested a regression model that included both guilt-NBE and gender as independent variables, but only found a significant main effect of guilt-NBE (guilt-NBE, *p* = 0.001; gender, *p* = 0.07; interaction, *p* = 0.08). We also did not find any significant differences between the different kinds of attributions (responsibility, causality, decision-making, and punishment), suggesting that these different attributions reflect a single underlying concept, moral culpability. We did not find any significant effects for any of the other tested variables. It should be emphasized however that the study was not designed specifically to test the effect of these demographic factors, and the sample size was likely not large enough to do so (particularly in the case of childhood and current religion).

## Discussion

The present studies contribute to the emerging literature on guilt and shame. The Moralizing Effect shows that individual proneness to guilt-affect, guilt-behavior, and shame-affect strongly predicts the severity of culpability attributions. Across the two experiments, it was demonstrated that increased proneness to guilt and affective shame (but not behavioral shame) significantly correlates with increased severity of culpability attributions of any type (i.e., responsibility, causality, decision-making, punishment). We found that this effect was robust across possible modulating factors.

Moreover, presenting experimental vignettes in the first- versus the third-person had no significant impact on attributional severity. This result is at odds with previous literature on the self-serving bias (Miller and Ross, 1975), which would predict more severe attributions about others than about oneself (Coleman, 2011). Our findings also contradict previous results about guilt and shame and attributions of causality and blame (Tangney, 1990; Tangney et al., 1992a,b), which would predict more severe attributions for guilt in the first person than for shame.

Furthermore, our results suggest that guilt- and affective shame-prone individuals are not moral hypocrites, i.e., those with a tendency to judge others more severely than themselves, as they tend to moralize in both first- and third-person culpability attributions. This result aligns with other studies showing that, even if moral hypocrisy is influenced by emotions, guilt is not among these emotions (Polman and Ruttan, 2011; Du et al., 2019). Our analysis also suggests that shame-affect is not linked to moral hypocrisy.

Finally, the introduction of factors that may mitigate culpability judgments did not influence the Moralizing Effect, despite evidence that such circumstances have historically been taken to mitigate criminal culpability in the legal domain (Hart, 1968) and despite the fact that these factors independently affected culpability judgments in our own data. As previously mentioned, the existing literature on guilt and shame favors the idea that guilt contributes to morality (in terms of pro-sociality) more than shame, but our results indicate a more nuanced interpretation of this view is required. Following philosophers like John Harris, we suggest that investigating prosociality is not enough to understand fully the morality of an emotion. For one, we found that not just guilt but also the affective dimension of shame strongly correlate with severe moral judgments about culpability. It follows that analyses of the morality of guilt should also consider the morality of affective shame. Guilt and shame-affect both function as indicators of the extent to which one holds oneself or others culpable for moral transgressions, with moralizing increasing, given greater proneness to these emotions. Furthermore, on the basis of the foregoing, one may want to reconsider the nature of guilt and shame (which are typically) characterized as “self-condemning,” in relation to emotions such as contempt, anger, and disgust (which are typically characterized as “other-condemning”) (for these classical distinctions see Haidt, 2003).

Some might argue that the moralizing effect we described is a positive sign of the morality of guilt and shame-affect prone people. This a plausible interpretation that has been suggested by previous literature so far (see Tangney et al., 2007). However, it is not clear that attributing a very high degree of culpability, especially to others (for some, this is called *moral condemnation*, see Cheng et al., 2013), is most consistent with “moral paths in life” as June Tangney believes. Indeed, we often speak colloquially of the vice of being too judgmental, harsh, or punitive. In other words, we tend to expect a “moral” agent to be measured, balanced or neutral when expressing judgments. If we think of guilt and shame in dimensional terms, we can infer that an excess of guilt and shame-affect produces outcomes we would not be willing to endorse if we want to be “moral.” We are open to the possibility, however, that perhaps people who are prone to guilt or shame-affect may have high moral standards for their own behavior, thus being moral with regard to themselves, while at the same time being too harsh, and therefore possibly immoral with regard to others. This interpretation would require further empirical investigation.

In other research domains such as, for example, disgust studies, moralization has been understood to have a negative connotation because it leads to or amplifies attitudes that we find socially undesirable (e.g., social prejudice) (see Rozin, 1999; Kelly, 2011). Because shame has been coupled with disgust in this literature (e.g., Giner-Sorolla and Espinosa, 2011; Terrizzi and Shook, 2020), it is also seen as generating negative outcomes (see also Nussbaum, 2009), while guilt is not (Clark and Fessler, 2015, p. 484 for a discussion). Without necessarily meaning that guilt and shame-affect make agents “immoral,” insofar as moralizing is a tendency towards being excessively punitive or intolerant, it is plausible to believe that they may lead to outcomes that are harmful or unjust. This means that the relationship between guilt or shame-affect and moralizing might not be a desirable one. Moralism, in fact, has been identified as a negative attitude because it contributes to making people “uncharitable and unsympathetic” (Archer, 2018).

Consistent with a Nietzschean perspective, moralizing may be considered a morally contestable behavior. In *Beyond Good and Evil* (Nietzsche, 1886/2006), Nietzsche claimed that “Assuming that … the oppressed, the suffering… should moralize: what would their moral valuations have in common? Probably a pessimistic suspicion towards the whole human condition would find expression, perhaps a condemnation of man together with his condition.” Instead of contributing to prosociality, for Nietzsche, moralization actually generates pernicious negative attitudes towards others.

Interestingly, proneness to shame-behavior (i.e., withdrawal), a trait that is intuitively associated with moral disentanglement or abdication of one’s own ethical responsibility, did not correlate with these moralizing attitudes in the first or the third person. A tentative interpretation of this finding is that proneness to withdrawal involves a waiving of the self-directed judgment, also preventing the individual from moral severity towards the other. These considerations may provide reasons to reconsider the classification of emotions such as guilt and shame, and their subtypes, as either simply “prosocial” or “anti-social.” Instead, each emotion may fall under both classifications concurrently, to different degrees that vary with contextual features.

## Limitations and future considerations

One limitation of this study is that in the GASP scale, like in previous scales, dispositional guilt and shame are assessed by self-reported responses to imaginary experiences. We did not investigate occurrent states of guilt and shame, nor how guilt and shame impact culpability attributions when they are elicited in the moment by real situations. As such, we encourage future research that induces guilt and shame in order to study other instances in which the Moralizing Effect might occur.

We did not include blame, which is conceptually distinct from responsibility, explicitly in our attribution questions (i.e., desert of punishment, volition, and causality). This means that conclusions about blame specifically cannot be drawn from these results. However, we speculate that the Moralizing Effect could be extended to judgments of blame, and suggest including this dimension in future study.

Even if excessive guilt and shame are associated with a series of psychopathologies, we cannot make any further assumptions about the relationship of the Moralizing Effect to psychopathological forms of guilt and shame. Our studies did not have the tools to test for the possible existence of psychopathological traits in our participants, nor did we establish normative criteria for a precise threshold for what counts as excessive guilt or shame.

We focused on the moralizing aspects of guilt and shame-affect, but it is also worthwhile to draw attention to the fact that proneness to withdrawal (shame-affect) did not appear to be a moralizing emotion, which is an interesting issue to explore and clarify in the future.

Some authors in the clinical literature (Basile et al., 2011; Mancini and Gangemi, 2021) have argued for the existence of two sub-forms of guilt, which are differentiated on the basis of which moral goals are transgressed: namely, “altruistic guilt” and “deontological guilt.” Altruistic guilt involves failing to put someone else’s good before one’s own (e.g., expressed by phrases like “How could I have hurt them so badly?” “How unfair! I am doing so well, while she/he is so unlucky!”), and deontological guilt involves the violation of an internalized moral norm (e.g., expressed by phrases like “How could I do such a thing?” or “How could I behave that immorally!”). When designing our study, we did not consider incorporating these forms of guilt, which assess other relevant aspects of moral affect and reasoning. It might be interesting to expand our research work in the future so as to understand the connections this distinction might have with the four dimensions of the GASP scale, as well as to clarify if and how the moralizing effect we found would be expressed when considering altruistic and deontological aspects.

We expected childhood religion or current religion to affect guilt and shame, and therefore our Moralizing Effect, but there was no significant impact. This may be because our sample was not sufficiently diverse with respect to religion, and so the sample size for each individual religion was too small for substantive analysis. For future research, we suggest recruiting larger samples from different religious faiths. Similarly, our analyses did not reveal any significant gender-related effects, but such effects may be revealed in future studies given a larger sample size.

In investigating the interaction between subcomponents of guilt and shame with moral attributions, this preliminary study offers a tentative “decomposition” of the functioning of these cognitive phenomena (Bechtel, 2002). From the perspective of integrative neuroscience, we hope that this may guide future neuroimaging research that explores the structures underlying these phenomena. Given the relevance of the notion of responsibility in various domains, the Moralizing Effect may have important ramifications for further areas of study and public policy. Some of these include educational and organizational psychology, regarding child development and professional responsibility, respectively; psychiatry and mental health, concerning the understanding of mental disorders; and ethics, law and politics, with their respective notions of moral, legal, and social responsibility.

## Conclusion

Evidence for the existence of The Moralizing Effect, which is the phenomenon that (both affective and behavioral) guilt and only affective (but not behavioral) shame are associated with the severity of culpability attributions of any type, is an important contribution to the psychological debate about self-directed emotions and responsibility. Contrary to the dominant narrative in psychology, which tends to favor the idea of the morality of guilt over shame, the Moralizing Effect calls into question the relationship between self-directed emotions and morality in a different way, permitting consideration of the moral/immoral character of these emotions from a dimensional perspective. The relationship we have found between moral emotions and responsibility attributions may have important ramifications for various areas of study and public policy.

## Data availability statement

The datasets presented in this study can be found in online repositories: https://figshare.com/s/22f87f1eabc918559479. The names of the repository/repositories and accession number(s) can be found in the article/supplementary material.

## Ethics statement

The studies involving humans were approved by Institutional Review Board at the City University of New York, IRB File #2015–1,353 issued on 11.30.2015. The studies were conducted in accordance with the local legislation and institutional requirements. The participants provided their written informed consent to participate in this study.

## Author contributions

ES conceived and designed the study, and drafted the article (except for the statistical parts). BA designed the vignettes in the study and realized the related tables. ES and JS did the data collection and organization. JS took care of permissions and communications. TW performed the statistical analyses, wrote the results section, including the realization of the relevant figures and tables. All authors together discussed, corrected and revised the design and the entire article, reviewed, discussed and together conceived the interpretation of the results, and approved the final version of the manuscript.
